# Exercise-Induced Shear Stress, Endothelial Glycocalyx Remodeling, and Atherosclerotic Plaque Stability: A Mechanistic Review

**DOI:** 10.3390/jcdd13060265

**Published:** 2026-06-12

**Authors:** Zihong Qi, Chenggang Zhang, Huilin Shi, Wen Li, Yuqing Xia, Xiaofeng Yan, Xiyan Zhou, Jiaqi Ling, Guochun Liu

**Affiliations:** 1 Department of Biomedical Engineering, Hong Kong Polytechnic University, Hong Kong 999077, China; qzh571915@163.com; 2 Sports Department, Harbin Institute of Technology, Harbin 150000, China; hitzcg@hit.edu.cn; 3 School of Sport and Health, Shenyang Sport University, Shenyang 110000, China; huiliniukoi77@163.com; 4 School of Public Health, Chongqing Medical University, Chongqing 400016, China; 2025223128@stu.cqmu.edu.cn (W.L.); 2025223129@stu.cqmu.edu.cn (J.L.); 5 The Second Clinical College, Chongqing Medical University, Chongqing 400016, China; 2024220852@stu.cqmu.edu.cn (Y.X.); 2025221004@stu.cqmu.edu.cn (X.Z.); 6 University of Leicester Joint Institute, Chongqing Medical University, Chongqing 400016, China; 2024221691@stu.cqmu.edu.cn; 7 College of Exercise Medicine, Chongqing Medical University, Chongqing 400016, China; 8 Division of Sports Science and Physical Education, Tsinghua University, Beijing 100084, China

**Keywords:** exercise training, wall shear stress, endothelial glycocalyx, endothelial barrier, vascular permeability, plaque stability

## Abstract

Acute cardiovascular events driven by atherosclerosis primarily originate from thrombosis triggered by vulnerable plaque rupture or endothelial erosion. Endothelial barrier destabilization—characterized by glycocalyx impairment, intercellular junction disassembly, and abnormal cytoskeletal tension—is a core upstream pathological stage that promotes atherogenic lipoprotein leakage, inflammatory cell infiltration, and matrix degradation. Hemodynamics, primarily through wall shear stress (WSS), shape the spatial distribution and plaque phenotypes of atherosclerosis; notably, low or oscillatory shear stress is associated with, and in experimental systems can promote, pro-inflammatory, pro-oxidant and pro-permeability endothelial phenotypes that contribute to plaque initiation and vulnerability. Conversely, regular exercise training, as an intervention that modulates hemodynamics, is widely suggested to promote anti-inflammatory, antioxidant, and antithrombotic endothelial phenotypes by significantly increasing antegrade shear stress and reducing detrimental retrograde/oscillatory shear stress. With a central focus on the axis of “exercise-shear stress-glycocalyx-cytoskeleton/junction-permeability-plaque stability,” this review integrates evidence from in vitro flow chambers, animal models and human studies to critically discuss: (1) the spatiotemporal heterogeneity of WSS and its relationship with plaque vulnerability; (2) the composition, barrier function, and plasticity of the glycocalyx as the primary interface for shear stress; (3) the mechanosensory complexes at the glycocalyx and junctions that transduce shear stimuli to protective pathways such as Phosphoinositide 3-kinase (PI3K)-Akt-endothelial nitric oxide synthase (eNOS) and Krüppel-like factor 2 (KLF2), thereby stabilizing adherens/tight junctions; (4) how improved barrier homeostasis promotes the maintenance of the fibrous cap collagen scaffold by reducing lipoprotein leakage and dampening the inflammation–matrix metalloproteinase (MMP) axis. Finally, this review highlights the boundary conditions of the biological effects of shear stress: low/oscillatory shear stress is primarily associated with plaque initiation and susceptible sites, whereas focal, extremely high WSS in established stenotic lesions may contribute to late-stage high-risk remodeling. Therefore, the protective hemodynamic adaptations induced by exercise should not be simply equated with the pathologically high WSS found at stenotic sites.

## 1. Introduction

Acute cardiovascular events caused by atherosclerosis are primarily driven by plaque rupture or endothelial injury complicated by thrombosis. Therefore, plaque stability, which is mainly determined by fibrous cap thickness, inflammatory burden, and matrix degradation, has emerged as one of the core pathological links dictating patient clinical outcomes [1,2]. In this evolutionary process, extracellular matrix degradation and fibrous cap thinning mediated by the protease system (especially matrix metalloproteinases, MMPs) are considered the key mechanisms driving the transition of plaques from a “stable” to a “vulnerable” state [3]. As the first physical and biological barrier between the blood and the arterial wall, the vascular endothelial layer plays a crucial role in maintaining the permeability homeostasis of the vascular microenvironment, finely tuning local inflammatory responses, and determining the morphological fate of atherosclerotic plaques [4]. When endothelial barrier function is compromised, atherogenic factors in the plasma more readily penetrate the endothelium and infiltrate the intimal layer, thereby inducing inflammatory cell infiltration and foam cell formation. This not only exacerbates lipid deposition and the expansion of the atheromatous plaque but also continuously amplifies the inflammatory response, ultimately propelling the plaque toward a high-risk vulnerable phenotype [5,6]. Conversely, an intact endothelial barrier can effectively restrict lipid entry and inflammatory mediator permeation from the luminal input, thereby attenuating plaque progression and preserving its structural stability.

As a classic non-pharmacological intervention, regular aerobic exercise has been well-documented to significantly promote cardiovascular health by optimizing the hemodynamic environment [7,8]. During exercise, the elevation in cardiac output and blood flow velocity continuously exposes the arterial endothelium to high-magnitude laminar shear stress; this physiological mechanical stimulus effectively drives endothelial cells to shift toward an anti-inflammatory, atheroprotective phenotype [9,10]. However, it is worth noting that the benefits of exercise in reducing atherosclerosis risk cannot be entirely explained by the amelioration of traditional systemic risk factors (e.g., blood lipids, blood pressure, and blood glucose). Mounting evidence supports that exercise itself acts as a potent mechanobiological stimulus, directly targeting and remodeling the vascular wall structure, thereby inducing anti-atherosclerotic adaptations at both functional and structural levels [11]. Exercise-induced blood flow shear stress not only dramatically upregulates endothelial nitric oxide (NO) production and improves vasodilation but also suggests a potential to attenuate the initiation and progression of atherosclerosis via a series of biomechanical pathways [12]. In this context, the repeated elevation of endothelial shear stress caused by increased blood flow during exercise is considered the key proximal stimulus triggering endothelial adaptation. In human exercise training studies, “clamping or manipulating shear” can significantly attenuate or even abolish the improvements in endothelial function, providing robust support for the causal contribution of shear stress [13]. Thus, exercise-induced shear stress, by reinforcing endothelial barrier function, holds promise as a critical mechanistic pathway for intervening in the evolution of atherosclerotic plaques [14].

Importantly, the efficacy of this exercise-mediated hemodynamic axis is not uniform across individuals; several key pathophysiological risk factors can substantially modulate the mechanosensory capacity of the endothelial glycocalyx interface, thereby conditioning the magnitude of exercise-induced vascular benefit. Specifically, chronic hyperglycemia and dyslipidemia impair endothelial glycocalyx (eGCX) integrity through sustained oxidative stress and glucotoxicity, creating a state of baseline mechanosensory deficit in which shear-induced protective signaling is attenuated [15]. Microvascular dysfunction—manifested as altered endothelial phenotype, capillary rarefaction, and impaired nitric oxide (NO) bioavailability—further limits the transmission of exercise-derived hemodynamic signals to the downstream barrier machinery. Additionally, the sub-intimal accumulation of partially oxidized lipids in early lesion-prone regions creates a pro-inflammatory microenvironment that primes the endothelium toward a pro-permeability state independent of shear input. These factors should therefore be understood not merely as background comorbidities, but as active moderators of the central mechanistic axis proposed in this review—namely, that exercise-induced shear stress may promote plaque stability via glycocalyx remodeling and barrier function restoration. Their presence defines the boundary conditions under which this mechanistic chain operates most efficiently, and underscores the need for personalized exercise prescriptions that account for individual metabolic and vascular status.

It must be emphasized that shear stress is not a singular metric where “higher is always better”; rather, its biological impact depends simultaneously on its magnitude, directionality, time-averaged level, and oscillatory characteristics [16]. In atherosclerosis-susceptible regions, such as arterial bifurcations and curvatures, low wall shear stress or oscillatory shear more frequently drives pro-inflammatory, pro-oxidant, and pro-permeability endothelial phenotypes, which aligns with the spatial distribution of early atherosclerotic lesions; conversely, in plaques where significant stenosis has already developed, focal, extremely high wall shear stress resulting from the geometric remodeling of the lesion especially when located proximal to the stenosis or at the plaque shoulder may be associated with endothelial injury, high-risk remodeling, and even plaque instability [17,18]. Therefore, the impact of shear stress on plaque evolution exhibits pronounced stage- and site-dependence: low/oscillatory shear is predominantly involved in plaque initiation and progression, whereas focal, extremely high WSS within the context of pathological stenosis may contribute to the formation of late-stage, high-risk phenotypes [19,20,21]. Based on these boundary conditions, the exercise benefits discussed in this review primarily refer to the exercise-induced, systemically recurrent favorable remodeling of shear waveforms, rather than the focal, extreme high WSS localized at pathological stenoses [13,14,21,22,23].

This review focuses on the mechanistic chain of “how exercise-induced shear stress may promote plaque stability through glycocalyx remodeling, endothelial junction/cytoskeleton coupling, and barrier function improvement,” with the ultimate aim of providing a more robust evidence framework for the mechanistic elucidation of exercise prescriptions and the identification of translational biomarkers.

## 2. Hemodynamic Characteristics of Exercise-Induced Shear Stress and Glycocalyx Responses

### 2.1. Heterogeneity of Shear Stress and Plaque Susceptibility

WSS, the tangential frictional force generated by blood viscosity on the vascular wall, serves as one of the most direct and continuous mechanical inputs to endothelial cells [24]. Its magnitude is typically expressed in dynes per square centimeter (dyn/cm^2^). Influenced jointly by vascular geometry and pulsatile blood flow, WSS exhibits significant spatial and temporal heterogeneity. Differences in vascular anatomy lead to pronounced variations in local shear stress patterns. In straight, uniform, and smooth arterial segments, blood flow predominantly presents as steady, unidirectional laminar flow, generating unidirectional WSS with a high average magnitude. Conversely, in complex flow-disturbed regions such as arterial bifurcations, curvatures, and proximal dilations, low-average or oscillating shear stress frequently occurs, exhibiting the characteristics of “disturbed flow” with repeatedly reversing or multidirectional patterns [24,25,26]. Extensive research demonstrates that such low/oscillatory shear environments are associated with pro-inflammatory, pro-oxidant, pro-permeability, and pro-remodeling endothelial phenotypes, closely aligning with the spatial distribution of early atherosclerotic lesions, which suggests their critical role in plaque initiation and progression [24,27]. This explains why atherosclerotic lesions predominantly occur at sites with low or disturbed blood flow shear stress (e.g., arterial bifurcations). In these susceptible regions, chronic and prolonged exposure to low shear stress induces endothelial dysfunction and exacerbates inflammatory responses, thereby promoting the initiation and progression of atheromatous plaques [28].

Furthermore, it is necessary to avoid an absolute dichotomy of “high/low shear stress” based on a single numerical threshold. Studies increasingly favor defining atheroprotective or atherogenic shear environments using a combination of metrics: directionality (unidirectional laminar vs. oscillatory/multidirectional), time-integrated magnitude (e.g., time-averaged WSS, TAWSS), and the degree of oscillation (e.g., oscillatory shear index, OSI), emphasizing that these parameters vary depending on the vascular bed, measurement techniques, and modeling methods. A shear environment characterized by unidirectionality, high TAWSS, and low OSI is generally associated with atheroprotective endothelial responses, including anti-inflammatory, antioxidant, and anti-adhesive states, as well as enhanced NO bioavailability. In contrast, low TAWSS coupled with high OSI or multidirectional shear often drives pro-inflammatory, pro-permeability, and pro-remodeling endothelial phenotypes, thereby establishing the microenvironmental foundation for lipid retention, immune cell infiltration, and subsequent plaque progression [29].

To ensure clarity and precision in extrapolating experimental findings to clinical pathophysiology, it is crucial to define the quantitative thresholds of these hemodynamic terms. The absolute values of “atheroprotective” versus “atherogenic” WSS vary significantly depending on the specific vascular bed, species, and whether the data originates from in vitro flow chambers or in vivo measurements. A summarized consensus of WSS quantitative thresholds referenced in this review is presented in Table 1.

### 2.2. Exercise Modalities and Shear Waveforms

From a sports physiology perspective, regular exercise provides a reproducible and quantifiable hemodynamic intervention for the arterial system. Regular exercise periodically generates high-magnitude pulsatile laminar shear stress stimuli throughout the systemic arteries, continuously exposing endothelial cells to a beneficial mechanical environment [9,30]. Exercise does not impose a uniform increase in shear across all vascular beds; rather, it reshapes shear waveforms in a vascular-bed-specific and time-dependent manner, often increasing antegrade and pulsatile laminar shear in arteries supplying active muscles while transiently augmenting retrograde components in non-working limbs. When the net hemodynamic shift favors high-magnitude, antegrade-dominant flow, this remodeled shear environment can trigger protective endothelial adaptations, including increased NO production, reduced oxidative stress, and the downregulation of inflammatory genes, thereby potentially counteracting the progression of atherosclerosis [31]. Dynamic aerobic exercise significantly elevates cardiac output and tissue perfusion, leading to an upregulation of shear stress characterized by augmented pulsatility in both the aorta and peripheral conduit arteries. This “repeated exposure” to a favorable shear environment is considered one of the critical proximal mechanisms by which exercise improves endothelial function and vascular adaptations [11,13,32,33]. In other words, exercise not only alters “shear magnitude” but also remodels “shear waveforms,” and the glycocalyx is situated at the very starting point of this mechano-biological signaling chain.

Notably, the exercise-induced shear waveform is not simply a “global elevation.” Its directional components can be divided into antegrade and retrograde shear, and their ratio and duration strongly depend on the exercise modality, exercise duration, and dynamic changes in downstream vascular resistance. For example, dynamic exercise involving large muscle groups (e.g., cycling, running) typically results in predominantly antegrade shear in the feed arteries of the working muscles with a significant increase in magnitude. Simultaneously, it generates complex shear responses in the conduit arteries of non-working limbs: during the initial phase of exercise, sympathetic vasoconstriction can transiently increase downstream resistance, leading to an augmentation of retrograde shear in non-working limbs. As exercise continues, thermoregulatory cutaneous vasodilation reduces downstream resistance, allowing retrograde shear to gradually decline and transition toward a waveform characterized by “predominantly antegrade/higher average shear” [33,34]. This time-dependent shear “waveform drift” indicates that the mechanical signals exerted on the endothelium by the same exercise prescription are not constant across different time windows.

Furthermore, different exercise modalities exert distinct shaping effects on shear waveforms. Continuous endurance exercise is more likely to create a relatively stable, antegrade-dominant shear environment, reinforcing the “time-integrated shear dose” through prolonged exposure. Interval training provides higher peak shear and stronger pulsatility within a shorter timeframe, potentially highlighting the threshold and saturation characteristics of mechanotransduction. Resistance or high-compressive exercises can alter instantaneous shear distribution through blood pressure fluctuations and temporary blood flow restriction, making the coupling of “shear and transmural pressure/strain” more prominent. Overall, conceptualizing exercise as a “programmable hemodynamic intervention” requires the simultaneous characterization of intensity (shear magnitude), duration (exposure time), and waveform (antegrade/retrograde/oscillatory ratio) [13,35]. More crucially, previous human validation studies have shown that experimental shear manipulation (e.g., attenuating local shear increases during training) significantly blunts the training adaptations of conduit arteries, suggesting that shear stress is not a mere “epiphenomenon” but a causal driver of vascular adaptation [13,32]. Therefore, exercise should be explicitly positioned as an “upstream intervention that alters shear waveforms and shear doses,” which downstream primarily acts on the glycocalyx—the luminal mechanical interface. Representative studies and related evidence are summarized in Table 2.

## 3. Composition, Barrier Function, and Shear Sensitivity of the Glycocalyx

### 3.1. The Glycocalyx as a Permeability Barrier and Mechanosensory Interface

Covering the luminal surface of endothelial cells, the endothelial glycocalyx (eGCX) is a highly hydrated, negatively charged polysaccharide-protein network composed of proteoglycans, glycoproteins, their glycosaminoglycan side chains (particularly heparan sulfate, chondroitin sulfate, and hyaluronic acid), and soluble plasma components. Through spatial sieving and charge repulsion, this structure restricts macromolecules and blood cells from closely approaching the endothelial surface, acting as the crucial “first line of defense” to maintain a state of low permeability and anti-adhesion. Concurrently, through its mechanical coupling with the membrane/cortical cytoskeleton, it translates blood flow shear inputs into intracellular signals, serving as the critical interface for endothelial mechanotransduction [24,43].

Under normal physiological conditions, an intact eGCX effectively prevents plasma macromolecules and immune cells from making direct contact with endothelial cells via its negative charge and spatial sieving effects, thereby preserving the low-permeability state of the endothelial barrier [23,44]. Reported eGCX thickness varies widely—from submicrometre values to several micrometres—depending on vascular bed, species, tissue preparation, and imaging method. In particular, whether microvascular PBR-based indices or direct structural measurements of conduit arteries are used accounts for much of this variance. Thus, PBR-derived glycocalyx indices should be interpreted as microvascular barrier surrogates rather than direct measurements of the glycocalyx overlying coronary or carotid plaques. In terms of barrier maintenance, glycocalyx integrity is closely correlated with solute permeability in both microvessels and conduit arteries. Glycocalyx impairment increases the transendothelial passage of macromolecules such as albumin and promotes the rolling and adhesion of inflammatory cells, thereby amplifying the vicious cycle of “increased endothelial permeability–inflammatory infiltration–further damage” [18,24]. It is worth emphasizing that the eGCX is among the first structures to directly sense blood flow shear stress; repeated application of high shear stress to the glycocalyx promotes its structural reinforcement and functional optimization. Conversely, low or disturbed shear stress can compromise glycocalyx structural integrity [23]. Regarding mechanosensation, multiple in vitro studies indicate that glycocalyx-associated glycosaminoglycans are essential for shear-induced NO release and endothelial morphological adaptation. For instance, hyaluronic acid-related pathways are involved in shear-induced NO generation; when glycocalyx integrity is insufficient, endothelial adaptive responses to laminar shear, such as alignment, migration, and contact inhibition of proliferation, are blunted [20,45,46]. However, in environments characterized by low or oscillatory shear stress, the structure and composition of the glycocalyx undergo significant alterations. Low shear stress downregulates the synthesis of the two major glycosaminoglycans within the glycocalyx—heparan sulfate and hyaluronic acid—and decreases the expression of various membrane-bound proteoglycans, glycoproteins, and adhered plasma proteins, leading to the thinning and shedding of the glycocalyx layer. The degradation of the glycocalyx is accompanied by an increase in endothelial permeability [25]. Studies utilizing in vitro flow chambers have demonstrated that sustained exposure to a relatively lower shear stress setting (e.g., 12 dyn/cm^2^, which was experimentally defined as “low” relative to the high-shear control in that specific model, despite falling within the physiological range in certain human vascular beds) for 24 h causes the glycocalyx of cultured endothelial cells to redistribute from the apical region toward the cell periphery; the apical coverage area of sialic acid components within the glycocalyx significantly decreases, while becoming relatively enriched at the intercellular junctions [47]. Upon removal of the flow shear stimulus, the glycocalyx gradually restores its uniform apical distribution [47]. This finding of reversibility highlights that the glycocalyx is exquisitely sensitive to changes in the mechanical environment and is capable of dynamic structural remodeling. In contrast, under static, no-flow conditions, the distribution of the eGCX shows no overt changes, indicating that specific low shear stress stimuli are the primary drivers of glycocalyx remodeling [25]. Furthermore, shear stress-mediated glycocalyx rearrangement is tightly coupled to endothelial mechanobiological responses. Normal endothelium with an intact glycocalyx exhibits typical adaptations—such as cellular alignment and contact inhibition of proliferation—under a flow shear stress of 15 dyn/cm^2^; however, prior enzymatic removal of the glycocalyx attenuates these beneficial responses [48]. This demonstrates that the glycocalyx plays a pivotal role in transmitting shear stress signals into the cell interior and inducing endothelial functional adaptations.

From a rigorous mechanistic perspective, the glycocalyx can be positioned as the upstream node in the “shear–barrier” coupling: on the one hand, it directly influences the hydrodynamic boundary layer and near-wall solute concentration distribution, dictating the physical properties of the barrier; on the other hand, via mechanotransduction, it governs the homeostasis of the endothelial cytoskeleton and junctional complexes, thereby indirectly modulating the opening and closing propensity of the paracellular pathway [24,49,50].

Furthermore, critical vascular bed-specific heterogeneity exists in eGCX biology that limits direct translational inference. The composition, density, and mechanosensitivity of the glycocalyx differ significantly between the microcirculation (where much of the clinical PBR data is derived) and large conduit arteries (the primary sites of atherosclerosis). Therefore, extrapolating findings regarding eGCX shedding from in vitro models or microvascular assessments directly to the macrovascular atherosclerotic milieu necessitates rigorous contextualization. Future studies must account for these spatial differences when interpreting the systemic benefits of exercise-induced shear stress on local plaque vulnerability.

### 3.2. Shear Stress-Driven Glycocalyx Remodeling

The glycocalyx is not a static structure but rather a “plastic interface” that is highly sensitive to hemodynamic stimuli, capable of rapid rearrangement, and subject to synthesis/degradation rebalancing over longer temporal scales. Within a short time window (hours), altering the shear environment can lead to the redistribution of the glycocalyx across the cell surface: for instance, in vitro flow experiments reveal that under specific shear conditions, the glycocalyx undergoes spatial rearrangement from the apical region toward the cell periphery, suggesting its capacity for rapid responsiveness to shear directionality and local stress distribution [20]. Over longer time windows (days to weeks), the net mass of the glycocalyx depends on the dynamic equilibrium among its synthesis, assembly, and degradation/shedding, with the shear waveform acting as a critical upstream variable dictating this setpoint [23,50].

The mechanisms underlying glycocalyx impairment in disturbed flow environments feature a parallel multi-pathway architecture: low or oscillatory shear promotes glycocalyx degradation via oxidative stress and inflammation-associated enzyme systems (e.g., hyaluronidase/heparanase-related pathways), which is accompanied by increased endothelial permeability. Notably, hyaluronidase-related mechanisms have been implicated in explaining the sequential linkage of “low shear–glycocalyx impairment–barrier disruption [14,51].” In contrast, directionally stable laminar shear is highly conducive to maintaining glycocalyx assembly and functional homeostasis, enabling the glycocalyx to achieve a positive feedback loop of “structural reinforcement–enhanced mechanotransduction efficiency–barrier stabilization” under mechanical loading [19,24,50].

Mapping the aforementioned evidence onto exercise prescriptions allows for a more testable articulation of “dose–response and time-window” dynamics. A single bout of acute exercise may induce transient fluctuations in glycocalyx-related circulating markers under varying conditions of intensity, thermal load, and circulatory stress. Regular training, however, is more likely to drive the glycocalyx to maintain or restore a more homeostatic structure-function coupling by repeatedly providing favorable shear waveforms alongside a sufficient time-integrated dose. The acute responses of glycocalyx-related indices to exercise are context-dependent, underscoring the necessity to simultaneously quantify covariates such as shear waveforms, thermal load, and dehydration in study designs to accurately interpret the magnitude and direction of the “exercise–glycocalyx” association [35]. Therefore, when discussing the mechanistic axis of “glycocalyx–cytoskeleton/junctions–permeability–plaque stability,” exercise should be characterized as an upstream intervention that simultaneously alters shear magnitude, directionality, and time-integrated dose. Furthermore, evidence synthesis must distinguish between the mechanistic focuses of acute exposure (minutes to hours) and chronic training adaptations (weeks to months) [13,19]. It should be noted, however, that most evidence linking laminar shear to glycocalyx structural reinforcement derives from in vitro flow chamber models; direct in vivo human data demonstrating that exercise-induced shear quantitatively increases glycocalyx thickness or mass at atherosclerosis-prone arterial sites remains limited [42].

## 4. Coupling Mechanisms of Glycocalyx-Mediated Mechanotransduction and Endothelial Barrier Execution

### 4.1. The Glycocalyx as the Interface for Shear Stress Sensation and Initial Transduction

Exercise does not merely increase average blood flow velocity; rather, by repeatedly altering the temporal and directional structure of blood flow shear stress, it remodels the hemodynamic waveforms to which the endothelium is exposed, serving as the upstream driver of endothelial phenotypic reprogramming [11]. However, blood flow shear stress does not act directly on the endothelial cell membrane. Instead, it is first absorbed, sieved, and translated by the glycocalyx network located at the outermost luminal surface, which thereby determines the “entry format” of mechanical stimuli into the endothelial signaling system [52]. Situated on the apical membrane of the endothelium, the glycocalyx assumes the primary function of mechanical load distribution, wherein the glycosaminoglycan network—predominantly composed of heparan sulfate—acts as a crucial filtering and amplifying interface before shear signals enter the cell [53]. The glycocalyx possesses the dual attributes of a permeability barrier and a mechanosensor, enabling it not only to influence the transendothelial passage of solutes but also to dictate the precision with which the endothelium recognizes flow field information [20].

Mechanistically, shear stimuli must first undergo mechanical coupling via the glycocalyx before integrating with intercellular junctional regions and the intracellular cytoskeleton; thus, glycocalyx integrity directly determines whether subsequent junctional complexes maintain stability or enter a state of remodeling [54]. Classical endothelial mechanobiology has linked the endothelial shear-sensing interface to junction-associated complexes, establishing a framework of continuous mechanical signal transmission from the luminal surface to intercellular junctions, which provides a basis for explaining the chain reaction of the glycocalyx–junction–cytoskeleton axis [22]. In atherosclerosis-susceptible regions, the endothelium undergoes flow pattern-driven transcriptional and phenotypic reprogramming. Consequently, under identical average shear magnitudes, different waveforms can exert completely opposite effects on the glycocalyx and the barrier [55]. At the microscopic response level, early shear loading can induce the spatial rearrangement of glycocalyx components within lipid rafts, wherein glypican-1-mediated clustering of heparan sulfate is a critical step for the localization and amplification of mechanical information [56]. Furthermore, glypican-1 exhibits pronounced “shear magnitude dependence”; its deficiency attenuates endothelial nitric oxide synthase (eNOS) activation under high shear conditions, illustrating that glycocalyx components not only sense the “presence or absence of shear” but also participate in encoding “shear quality” [57]. Therefore, whether exercise-related blood flow stimuli are successfully translated into protective endothelial signals depends primarily on glycocalyx integrity and the availability of the apical mechanosensory unit consisting of glypican-1 and heparan sulfate [58].

### 4.2. Cytoskeletal Reorganization and Tension Redistribution Driven by Glycocalyx Remodeling

**Figure 1:**
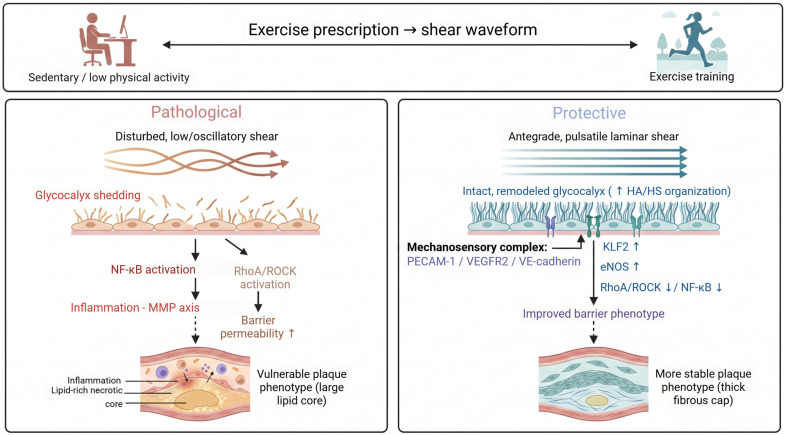
Proposed exercise–shear–glycocalyx pathway in atherosclerotic plaque stabilization.

The critical aspect of glycocalyx remodeling lies not in the thickness alteration itself, but in its modification of the load distribution pathways following luminal force application, shifting more of the tangential forces—originally buffered by the glycocalyx—onto the membrane cytoskeleton and junctional complexes [21]. When glycocalyx components are lost or spatially disorganized, the physiological transduction efficiency of shear stimuli by the endothelium declines. Mechanical signals are then more readily projected onto the cortical actin network in a pro-contractile, pro-disruptive manner, rather than being dissipated to maintain a homeostatic barrier. This mechanical redistribution propels vascular endothelial cadherin (VE-cadherin)-associated adhesions from a relatively stable state into a high-tension remodeling state, accompanied by enhanced radial F-actin pulling, thereby transitioning the junctional region from “closed” to more prone to opening [59]. In endothelial cells, force-dependent junctional remodeling is tightly coupled with the RhoA/Rho-associated protein kinase (ROCK)-myosin tension axis; hence, glycocalyx damage is not an isolated event but an upstream amplifier that escalates the propensity for junctional rupture [60]. Therefore, glycocalyx remodeling and cytoskeletal reorganization should be viewed as sequential phases of the same continuous process, rather than as two mutually independent pathological events. The stability of the endothelial barrier hinges not merely on the expression levels of individual junctional proteins, but fundamentally on whether the coupling among VE-cadherin, p120-catenin, β-catenin, α-catenin, and cortical actin maintains a continuous, force-controllable junctional complex [61]. The dual mechanistic pathways described in Section 4.1 and Section 4.2—glycocalyx-dependent mechanosensory activation under laminar shear, and RhoA/ROCK-driven barrier disruption under disturbed shear—are visually integrated in Figure 1.

Exercise prescription modulates vascular hemodynamics in a vascular-bed-specific manner, shifting the shear environment from a sedentary, low-activity state toward one characterized by antegrade, pulsatile laminar shear. (Left, Pathological) Disturbed or low/oscillatory shear promotes glycocalyx shedding, driving two parallel maladaptive cascades: NF-κB activation, which fuels the inflammation–MMP axis, and RhoA/ROCK activation, which increases barrier permeability. Together, these converge on a vulnerable plaque phenotype characterized by a large lipid-rich necrotic core and macrophage infiltration. (Right, Protective) Exercise-associated laminar shear supports intact glycocalyx remodeling (↑ HA/HS organization), activating the PECAM-1/VEGFR2/VE-cadherin mechanosensory complex. Downstream, KLF2 and eNOS are upregulated while RhoA/ROCK and NF-κB are suppressed, collectively promoting an improved barrier phenotype that may contribute to a more stable plaque phenotype over time. Solid arrows indicate relationships supported by experimental evidence from in vitro or animal studies; dashed arrows indicate inferred or incompletely validated links, particularly in the context of direct human exercise studies.

Abbreviations: eGCX, endothelial glycocalyx; HA, hyaluronic acid; HS, heparan sulfate (a glycosaminoglycan side chain of syndecan-1/syndecan-4 and glypican-1); PECAM-1, platelet endothelial cell adhesion molecule-1; VEGFR2, vascular endothelial growth factor receptor 2; VE-cadherin, vascular endothelial cadherin; KLF2, Krüppel-like factor 2; eNOS, endothelial nitric oxide synthase; NF-κB, nuclear factor kappa B; RhoA, Ras homolog family member A; ROCK, Rho-associated protein kinase; MMP, matrix metalloproteinase; SMCs, smooth muscle cells.

### 4.3. Junctional Complex Reprogramming and Increased Paracellular Permeability

Intercellular junctions in endothelial cells are not static physical seals; rather, they constitute an adjustable barrier system that integrates adhesion, tension sensing, and signal regulation. Therefore, “junctional integrity” is not entirely equivalent to “barrier effectiveness” [62]. Within this system, adherens junctions and tight junctions collectively determine the opening threshold of paracellular channels, a threshold that is continuously co-regulated by cytoskeletal tension, the local aggregation state of molecules, and the inflammatory signaling context [63]. The VE-cadherin complex is situated at the center of this system; its coupling state with β-catenin, α-catenin, and the actin cytoskeleton determines the junction’s capacity to resist leakage [59]. Under disturbed flow or pathological low shear conditions, the aberrant activation of β-catenin signaling occurs in parallel with the remodeling of junctional/adhesive structures, leading to the synchronized progression of increased permeability and amplified inflammation. This suggests that the reprogramming of the junctional complex serves as the critical hub mediating the endothelial transition toward a “pro-inflammatory and pro-leakage” vulnerable phenotype [64].

Regarding specific molecular execution mechanisms, the platelet endothelial cell adhesion molecule-1 (PECAM-1)–Nck1–PAK2 signaling axis is directly involved in the process of junctional rupture. Notably, Nck1 deficiency significantly inhibits shear-induced PAK2 membrane localization, paracellular pore formation, and fibrinogen extravasation into the vascular wall [65]. Furthermore, the tight junction protein Claudin-5 is not a passive receptor; its deletion is accompanied by a downregulation of ZO-1 and compensatory changes in VE-cadherin, ultimately compromising barrier robustness [66]. When inflammation and mechanical abnormalities are superimposed, nuclear factor kappa B (NF-κB)-dependent ROCK-MLC phosphorylation disrupts the colocalization of claudin-5 and cortical actin, shifting cytoskeletal tension from “peripheral support” to “contractile tearing,” thereby amplifying paracellular leakage [67]. Although mechanosensitive molecules at the junctional region (e.g., vinculin-related mechanisms) can enhance junctional resistance to rupture within a certain tension range, this compensation is highly dependent on intact junction-cytoskeleton coupling and homeostatic upstream inputs from the glycocalyx and the flow field [68]. Ultimately, once the junctional complex enters a state of persistent reprogramming, elevated paracellular permeability ceases to be a transient response and becomes an integral part of the endothelial phenotype in susceptible vascular segments, subsequently forming a positive feedback loop with lipid deposition and inflammatory infiltration [69].

### 4.4. Shear Pattern-Dependent Integration of Key Signaling Axes

The determinant of atherosclerosis-related endothelial alterations is not the mere “presence or absence of shear stress,” but rather its directionality and oscillatory nature; consequently, different flow waveforms dictate the entry of endothelial cells into distinct signaling trajectories [28]. Protective flow fields tend to maintain endothelial homeostasis and an anti-inflammatory phenotype, whereas disturbed flow drives endothelial dysfunction and pro-atherogenic reprogramming. This divergence constitutes the fluid dynamic premise of the glycocalyx-barrier mechanistic chain [69]. Mediated by steady flow and an intact glycocalyx, HEG1 undergoes lateral membrane relocalization in the endothelium. It subsequently upregulates Krüppel-like factor 2 (KLF2)/Kruppel-like 4 (KLF4) via the KRIT1-regulated MEKK3–MEK5–ERK5–MEF2 pathway, thereby suppressing permeability and the propensity for monocyte adhesion and migration [70]. The critical importance of this axis lies in its direct translation of “flow patterns” into transcriptional programs. KLF2/KLF4 are not merely isolated biomarkers but upstream master regulators that maintain the anti-inflammatory, anti-leakage, and anti-atherogenic state of the endothelium. Furthermore, high-magnitude laminar shear stress can rapidly activate the Phosphoinositide 3-kinase (PI3K)-Akt kinase cascade at the post-translational modification level, promoting eNOS phosphorylation and robust NO release. This dual-track transduction paradigm—paralleling transcriptional (KLF2) and kinase (PI3K-Akt) mechanisms—represents a well-supported protective axis in experimental settings, though its direct activation by exercise-induced shear in human vascular tissue remains to be confirmed in vivo. Conversely, the upregulation of γ-protocadherins in a pro-atherogenic environment can inhibit KLF2/KLF4 signaling, shifting the endothelium from a homeostatic program toward an inflammatory one. This suggests that the flow-sensitive transcriptional network itself represents a potentially modifiable mechanosensitive regulatory node [71]. However, it remains debated whether the transient, intermittent spikes in shear stress experienced during typical human exercise training are sufficient to chronically sustain these mechanosensory transcriptional programs (such as KLF2 upregulation). Most direct causal evidence for these pathways is derived from in vitro continuous flow models, which may not perfectly replicate the complex, pulsatile, and resting-phase hemodynamics of in vivo human exercise. The value of exercise at this level resides in its ability to remodel the shear inputs received by the endothelium through repetitive, rhythmic hemodynamic pulses, thereby altering the subsequent execution state of the barrier [11]. Different exercise modalities induce distinct peripheral blood flow and shear waveforms, which dictate the proportional exposure of the endothelium to antegrade versus retrograde/oscillatory shear; this is a key reason underlying the heterogeneity of training adaptations [72]. Human studies have confirmed that localized attenuation of the shear stimulus during training significantly blunts endothelial functional adaptations, directly demonstrating a causal relationship between the mechanical stimulus and exercise benefits [13]. Simultaneously, because an increase in retrograde and oscillatory shear loads adversely affects endothelial function, it implies that “exercise efficacy” is predicated on the predominance of favorable shear waveforms; not every increase in blood flow equates to vascular protection [36]. This also explains why the effects of exercise are highly dependent on the “quality of the shear pattern” rather than merely elevated heart rate; only exercise workloads capable of increasing the proportion of steady antegrade shear are likely to drive the HEG1–KLF2/KLF4 axis toward a protective direction [11]. At the same time, mechanistic boundary conditions must be retained: certain high-intensity or high-volume acute exercise loads can induce transient elevations in syndecan-1 and heparan sulfate, indicating that the acute stress phase may be accompanied by shear-induced glycocalyx shedding, and that long-term training adaptations are not equivalent to acute responses [53]. Representative evidence for the above is summarized in Table 3.

## 5. Alterations in Endothelial Permeability and the Stable Phenotype of Atherosclerotic Plaques

### 5.1. Endothelial Hyperpermeability, Lipid Deposition, and Inflammatory Cell Infiltration

Pathologically, endothelial hyperpermeability should not be simplified as a singular, passive “physical leak,” but rather as a complex reprogramming of transmembrane transport pathways, manifested by a synchronous shift toward a decreased opening threshold of paracellular channels and enhanced transcellular transport [54]. This transport reprogramming dramatically increases the flux of pathogenic lipoproteins into the intima and amplifies the exposure of local tissues to inflammatory signals, thereby accelerating the transition of susceptible vascular segments into the coupled phase of lipid retention and immune cell recruitment [74]. Under protective laminar flow, the endothelium can secrete factors that inhibit transcellular transport and inflammatory effects, indicating that physiological flow fields can directly constrain early plaque “feeding” and inflammatory amplification [75]. Conversely, in regions of low shear stress or disturbed flow, barrier execution skews toward a pro-permeability state, where lipid deposition and inflammatory cell infiltration are no longer merely parallel events but a mutually driving continuous reaction chain [69]. Therefore, elevated endothelial permeability is not an incidental phenomenon following plaque formation, but one of the prerequisites driving the evolution of plaque components toward highly inflammatory and necrotic tendencies [76].

In this pathological chain, regular exercise is not a downstream “intervention variable”; rather, by repeatedly increasing blood flow and favorable wall friction, it drives the functional and structural adaptation of endothelial permeability to shear stimuli [11]. This upstream effect possesses distinct waveform attributes: training effectively increases antegrade shear and reduces retrograde shear and the oscillatory index, thereby shifting the mechanical environment of the local endothelium toward a more atheroprotective state [37]. The reason this “shear-training effect” can enter the atherogenic chain is crucially that endothelial permeability itself is a plastic mechanobiological phenotype, rather than a static barrier state [21]. The first stop for transmitting exercise signals to the permeability phenotype remains the glycocalyx, because heparan sulfate proteoglycans within the glycocalyx directly participate in shear sensation and NO-related mechanotransduction, determining the quality of the endothelial response to flow stimuli. When the shear environment favors high-magnitude laminar flow, the luminal glycocalyx tends to thicken and adopts an anti-inflammatory, anti-leakage state; conversely, oscillatory flow compromises glycocalyx integrity and reduces its barrier efficacy, a process closely linked to KLF2-regulated glycometabolic redistribution and HAS2 membrane localization [21]. Therefore, exercise-induced favourable shear waveforms may support the maintenance or restoration of a more protective glycocalyx configuration, with the KLF2–HAS2–hyaluronic acid axis serving as a potential molecular handle [21]; however, while the exercise-induced shear pattern remodeling that enables this shift has been demonstrated in human training studies [37], direct evidence at local atherosclerotic plaque sites in humans remains limited.

Exercise-related glycocalyx data should be interpreted cautiously. Acute exercise can alter circulating glycocalyx-shedding markers, including syndecan-1 and heparan sulfate, but increases in these markers do not by themselves indicate improved glycocalyx integrity [14]; they may reflect transient shedding, turnover, hemodilution, thermal stress or sampling-time effects. In contrast, repeated training appears more compatible with preservation or restoration of glycocalyx-related barrier properties in selected human and experimental settings. In metabolic disease, hyperglycemia, oxidative stress and inflammation can impair eGCX structure and may blunt shear sensing. Therefore, the exercise–shear–glycocalyx axis should be presented as a biologically plausible and testable mechanism rather than as a proven causal pathway linking exercise to plaque stabilization. When glycocalyx-shear transduction is mismatched, endothelial hyperpermeability amplifies the pathological process along two pathways: one is the increased opening of paracellular pathways caused by junctional complex destabilization (as previously discussed), and the other is the more targeted, aberrantly enhanced transcellular transport of low-density lipoprotein (LDL) [77]. Although direct evidence linking exercise-induced shear to these transcellular receptors remains limited, their mechanistic relevance to the permeability chain warrants consideration. This transcellular transport is not passive leakage; activin receptor-like kinase 1 (ALK1) can mediate LDL uptake and transendothelial transport, while scavenger receptor class B type 1 (SR-B1) promotes LDL endocytosis and transcytosis by recruiting dedicator of cytokinesis 4 (DOCK4) and activating RAC1, and these receptors are upregulated even before lesion formation [73]. This implies that lipid infiltration does not require waiting for “complete barrier rupture,” but occurs preemptively in the form of rewritten endothelial active transport programs under an abnormal shear context [74]. Concurrently, NF-κB signaling in the endothelium of susceptible sites is in a pre-activated state, making adhesion molecules and chemotactic programs more easily triggered, thereby coupling lipid entry and leukocyte infiltration into a unified pathological process [77].

The critical value of exercise intervention lies in that it does not merely act only at the terminal inflammatory stage, but simultaneously suppresses the gain of both pathways further upstream by remodeling the shear environment. For example, protective flow induces the endothelial secretion of follistatin-like 1 (FSTL1), which can synchronously reduce the transendothelial transport of LDL and its subfractions, and inhibit tumor necrosis factor-alpha (TNF-α)-induced NF-κB nuclear translocation, vascular cell adhesion molecule-1 (VCAM-1)/intercellular adhesion molecule-1 (ICAM-1) expression, and monocyte adhesion. This indicates that permeability control and inflammation control are mechanistically coupled processes at the level of flow signaling [78]. Accordingly, regular exercise is essentially a repetitive mechanical preconditioning applied to the vascular endothelium. Through shear-dependent glycocalyx maintenance, transport program constraint, and inflammation threshold elevation, it reduces LDL retention and immune cell entry, thereby creating the initiating conditions for plaque evolution toward a stable phenotype [37]. Although exercise can stabilize established plaques accompanied by systemic anti-inflammatory effects [79], it must be rigorously noted that direct causal evidence of exercise explicitly regulating the ALK1 or SR-B1–DOCK4 axes remains scarcer than evidence for “exercise alters shear” and “shear controls transport.” Therefore, the most robust current articulation is that while the upstream mechanistic chain is relatively complete, exercise-specific validation at key nodes still requires further reinforcement at the local lesion scale [13].

### 5.2. Key Phenotypic Differences Between Stable and Vulnerable Plaques

From a pathological phenotype perspective, plaque stability fundamentally depends on the mechanical and biological balance between the load-bearing capacity of the fibrous cap and the necrotic core/inflammatory burden, rather than being solely dictated by plaque volume [76]. The pathological core of a vulnerable plaque is characterized by an expanded necrotic lipid core, a thinned fibrous cap, sparse smooth muscle cells, and macrophage enrichment; among these, the thin-cap fibroatheroma is the precursor lesion with the highest risk of triggering acute rupture. Classical pathological and imaging studies consistently confirm that fibrous cap thickness, inflammatory cell density, and necrotic core structure serve as key discriminant criteria: stable plaques lean toward fibrosis and structural encapsulation, whereas vulnerable plaques exhibit high inflammatory activity and structural fragility [1,80]. This implies that interventions targeting the endothelial barrier, even if they cannot rapidly reduce total plaque burden, can still significantly remodel plaque composition by altering the input flux of lipids and inflammatory cells [81]. The alteration of plaque fate by endothelial hyperpermeability is primarily manifested by an increased input burden. Enhanced LDL transcytosis and inflammatory cell infiltration jointly drive foam cell formation and necrotic core expansion, amplify the local cytokine network, promote the expression of matrix-degrading enzymes, and ultimately compromise the collagen stability of the fibrous cap. Thus, abnormal endothelial permeability is not a “prelude” to early events, but an upstream driver that continuously shapes plaque composition and mechanical vulnerability [74]. It is necessary to clarify that the aforementioned key discriminant criteria for vulnerable versus stable plaques are mainly derived from clinical pathology and imaging consensus, whereas current causal validation evidence regarding exercise interventions reversing or directly stabilizing plaques remains predominantly based on animal models.

The value of exercise in animal models lies in reversing the trajectory of plaque evolution from a “high-input, high-inflammation, high-degradation” track to a “low-input, low-inflammation, high-repair” track; thus, its primary effect is often reflected in inflammation attenuation and compositional optimization, rather than a simple linear reduction in plaque area [82]. For instance, studies in ApoE-deficient mice demonstrate that exercise training not only reduces plaque area but also increases plaque collagen and elastin content, decreases macrophage infiltration and MMP-9 levels, and elevates tissue inhibitor of metalloproteinases-1 (TIMP-1). These changes do not depend on significant systemic lipid-lowering, suggesting its primary action site is the vascular wall microenvironment rather than merely lipid metabolism [83]. In diabetic atherosclerosis models, this stabilization effect further manifests as fibrous cap thickening and reduced internal elastic lamina rupture, occurring synchronously with decreased intra-plaque interleukin-6 (IL-6), MMP-2, MMP-3, and MMP-8, and increased TIMP-2. This indicates that exercise does not solely alter systemic metabolism but resets the inflammation-protease network within the lesion microenvironment [81]. Furthermore, recent studies point out that exercise can regulate the matrix degradation balance via exosomal miRNAs, evidenced by the downregulation of let-7c-5p and upregulation of TIMP-3, accompanied by decreases in MMP-9, IL-6, and TNF-α, thereby providing more direct evidence for the molecular pathways by which “exercise influences plaque composition [84].” Therefore, under exercise intervention, the “stabilization” of plaques can be reframed as an intervenable molecular phenotypic transition: shifting from the high-permeability-driven lipid–inflammation-protease coupling toward a low-rupture-risk state characterized by barrier maintenance, TIMP dominance, and fibrous cap reinforcement. It is also worth noting that vascular wall remodeling in this context is not solely dependent on resident endothelial cells; the exercise-induced mobilization of endothelial progenitor cells (EPCs) from the bone marrow represents an additional regenerative dimension. Exercise-induced alterations in vascular shear stress, through increases in blood flow and eNOS-derived NO production, act as a potent stimulus for EPC release from the bone marrow and their homing to sites of endothelial injury, where they contribute to barrier restoration and may participate in fibrous cap maintenance [85,86].

### 5.3. The Integrated Mechanistic Chain from Local Flow Patterns to Plaque Stability

Local flow patterns initially dictate the mechanical environment of the endothelium; protective laminar shear tends to maintain glycocalyx structure and mechanosensory capacity, whereas disturbed shear drives glycocalyx thinning, inflammatory susceptibility, and transport program imbalance. Subsequently, endothelial signals are reprogrammed at the level of junctional complexes and cytoskeletal tension, and are further projected onto the dual execution pathways of paracellular permeability and transcellular LDL transport, ultimately altering lipid entry, leukocyte adhesion/migration, and the intra-plaque inflammation-matrix balance from the source [21].

The protective effects of regular exercise can be attributed to its role as an upstream regulator capable of repeatedly applying “protective mechanical stimuli” to the endothelium: exercise remodels hemodynamic inputs, hemodynamic inputs remodel the glycocalyx and endothelial mechanotransduction, mechanotransduction shapes the barrier execution state, and this is proposed to ultimately influence the evolutionary trajectory of plaque components [55]. The key node in this chain is not a single isolated molecule, but the multi-layered coupled system of “flow pattern—glycocalyx integrity—junction/cytoskeleton tension—permeability pathway selection”; therefore, mechanistic explanations should emphasize system-level coupling rather than single-point attribution [20]. In the context of exercise, recurrent favorable shear stimuli provide a plasticity window for the endothelium, making it easier to maintain homeostatic programs of anti-inflammation, anti-leakage, and anti-adhesion, thereby reducing the probability of the plaque shifting toward a vulnerable phenotype [69]. This is consistent with evidence that human vascular adaptation is highly dependent on shear stress, and aligns with the framework that exercise-induced improvements in vascular wall health are primarily triggered by hemodynamic stimuli [11]. Regular exercise promotes adaptive endothelial remodeling by periodically increasing hemodynamic stimuli; this adaptation is reflected not only in improved vasodilation but also in the resetting of transport and inflammatory signals. FSTL1 secretion induced by protective flow can inhibit LDL transport and inflammatory activation, providing an integrative mechanistic pivot for how exercise shear signals simultaneously impact “lipid input” and “inflammatory amplification” [11]. Extending further downstream, exercise-related evidence of plaque stabilization has already shown molecular targets consistent with this chain, including the regulation of RhoA/ROCK-related pathways, restoration of the MMP/TIMP balance, downregulation of inflammatory factors, and increased collagen deposition. These alterations collectively point to enhanced mechanical strength of the fibrous cap and restricted expansion of the necrotic core. For this very reason, a more accurate articulation of the “exercise benefit” in atherosclerosis is the remodeling of plaque biology rather than merely the reversal of plaque burden [87].

In animal models of atherosclerosis, treadmill training can mitigate vascular inflammation and ameliorate adverse plaque-related signaling axes, illustrating that exercise acts not only through systemic metabolic changes but can directly intervene in the vascular wall microenvironment [84]. Meanwhile, exercise-induced anti-inflammatory effects and plaque stabilization are also observable in diabetic atherosclerosis models, further supporting the explanation of a “synergistic remodeling of barrier and inflammation” rather than a “single-factor lipid-lowering” pathway [81]. Currently, the weakest link in the evidence chain remains the direct in vivo human verification of the congruent trajectory encompassing glycocalyx remodeling, endothelial permeability alterations, and the plaque stabilization outcome. Therefore, future studies need to integrate local flow fields, glycocalyx imaging/biomarkers, barrier phenotypes, and plaque composition endpoints within the same study design framework [55]. Representative research evidence supporting the impact of exercise on downstream lesion burden and plaque phenotypic remodeling is summarized in Table 4.

## 6. Controversial Points and Discussion

Crucially, while the proposed “exercise–shear–glycocalyx–plaque stability” axis provides a highly plausible translational framework, the hierarchy of current evidence requires precise delineation. Human studies robustly demonstrate that exercise improves flow-mediated dilation (FMD) [13,37]; however, acute FMD changes across different exercise modalities can sometimes exhibit minimal statistical divergence (e.g., Lyall et al., 2019 [39]). Importantly, while the association between exercise and FMD improvement is well-established at the macrovascular level, the specific inference that this benefit is mechanistically mediated through the shear-glycocalyx-permeability pathway remains to be directly validated in humans. Thus, the current framework synthesizes parallel lines of evidence—juxtaposing macroscopic human vascular benefits with microscopic in vitro and animal models—rather than a single, unbroken causal chain. Rather than invalidating the hypothesis, this scarcity of simultaneous in vivo human assessments highlights a critical translational frontier. Future clinical trials must concurrently quantify shear waveforms, glycocalyx dimensions, and plaque composition to fully cement this causal continuum.

Although the aforementioned sections have emphasized the stage- and site-dependence of shear stress effects, the high-risk remodeling mechanisms mediated by focal, extremely high WSS in the context of late-stage lesions remain a key area worthy of further investigation. Current research suggests that the extremely high WSS proximal to severe stenoses or at plaque shoulders may be associated with endothelial injury, intra-plaque neovascularization, and destabilization. This indicates that “favorable shear” and “pathologically high WSS” should be understood separately across different disease stages and should not be lumped into the same protective framework.

As introduced above, metabolic disorders including diabetes and hyperlipidemia maintain the eGCX in a state of chronic impairment. In such patients, persistent oxidative stress and glucotoxicity resulting from systemic inflammation and hyperglycemia keep the eGCX in a continuous state of impairment or shedding [15]. Under these circumstances, exercise-induced shear stress signals may struggle to be effectively transduced through the compromised glycocalyx, thereby blunting the endothelial protective effects of exercise. This also explains certain clinical observations where aerobic exercise yields less pronounced improvements in endothelial function in some diabetic patients compared to healthy individuals, potentially attributable in part to decreased mechanosensitivity resulting from glycocalyx damage. Nevertheless, long-term regular exercise itself has been found to ameliorate metabolic profiles and reduce inflammation, thereby indirectly promoting glycocalyx repair. In human training studies, long-term moderate-intensity endurance exercise has been shown to reduce circulating glycocalyx damage markers, including syndecan-1 and heparan sulfate, accompanied by attenuation of oxidative stress, suggesting that regular training promotes glycocalyx structural homeostasis [40]. Furthermore, in diabetic atherosclerotic animal models, exercise training significantly reduced local inflammatory burden and improved plaque composition [81], further supporting the rationale that progressive exercise interventions may partially restore glycocalyx function. Therefore, even if the glycocalyx is initially damaged, progressive exercise interventions may still partially restore its structure and function, thereby rebuilding endothelial mechanosensory capacity. To this end, some scholars have proposed combining pharmacological approaches (e.g., using glycocalyx-protecting agents like sulodexide) to enhance the positive effects of exercise on the glycocalyx. Future prospects may see the integration of exercise prescriptions with pharmacological or nutritional interventions as comprehensive strategies to maximally improve eGCX and barrier function.

The differential impacts of acute versus chronic exercise on the glycocalyx also warrant consideration. While a single bout of intense exercise may transiently elevate plasma glycocalyx shedding markers—likely reflecting physiological turnover rather than permanent damage—long-term regular training consistently drives the glycocalyx toward a more homeostatic and protective configuration. These findings underscore the importance of sustained exercise adherence over isolated bouts, and highlight the need to account for exercise intensity and individual baseline status when interpreting glycocalyx responses.

Furthermore, a significant limitation in the current literature—and consequently in this review—is the absence of a quantitative dose–response synthesis regarding exercise and glycocalyx health. While qualitative differences between exercise modalities (e.g., continuous endurance versus high-intensity interval training) have been observed, the precise quantitative thresholds of exercise intensity, optimal session duration, and weekly frequency required to maximize the protective shear-glycocalyx axis remain undefined. Presenting a standardized clinical exercise prescription is currently precluded by the heterogeneity of measurement techniques and vascular bed-specific responses. Therefore, future studies must systematically titrate exercise variables to establish these missing quantitative clinical guidelines.

Moreover, the potential heterogeneity of this mechanistic axis across different age and sex groups warrants consideration. Aging is inherently associated with endothelial dysfunction and reduced glycocalyx mechanosensitivity, which may attenuate the vascular adaptations to exercise-induced shear stress commonly observed in young, healthy populations [38]. Similarly, sex hormones can independently influence glycocalyx integrity and endothelial responses. Because the primary clinical demographic for atherosclerosis management comprises older adults with comorbidities, future research must specifically elucidate how age and sex modulate the efficacy of the “exercise–shear–glycocalyx” axis.

Additionally, in real-world clinical scenarios, exercise is typically prescribed as an adjunct to pharmacological therapies, such as statins, antihypertensives, and antiplatelet agents. Notably, statins are known to independently upregulate KLF2 and enhance eNOS bioavailability, potentially exerting synergistic protective effects on the eGCX when combined with exercise-induced shear stress. While the specific interactions between these standard medications and the exercise-glycocalyx axis remain largely unexplored, integrating hemodynamic interventions with pharmacological profiles represents a critical avenue for optimizing personalized cardiovascular rehabilitation.

Finally, from a translational medicine perspective, monitoring and evaluating the status of the glycocalyx-endothelial barrier axis presents a major challenge. Several clinical methods for assessing the glycocalyx have been developed, such as indirectly inferring glycocalyx thickness by measuring the perfused boundary region (PBR) of red blood cells in the microcirculation. Some studies suggest that glycocalyx damage could serve as an early marker for cardiovascular disease. However, results from cross-sectional studies are not entirely consistent; a multi-ethnic cohort study found that reduced glycocalyx thickness was significantly associated with female sex and diabetes, but its independent association with overall cardiovascular disease incidence was not strong. This reminds us that the clinical significance of glycocalyx-related indices requires further clarification. Perhaps future approaches will require combinatorial metrics, such as integrating glycocalyx damage markers with endothelial function tests (e.g., FMD, flow-mediated dilation), to more accurately reflect the health status of the “glycocalyx-barrier” axis. In this domain, exercise intervention trials can provide additional insights. By comparing the changes in glycocalyx indices and plaque imaging characteristics before and after exercise, the applicability of this mechanistic chain in humans can be verified. If it can be confirmed that exercise promotes plaque stability via improving the glycocalyx and endothelial barrier, glycocalyx-related metrics could emerge as useful clinical tools for evaluating exercise efficacy and cardiovascular risk reduction.

## 7. Conclusions and Perspectives

In summary, extensive basic and preclinical studies support the following proposed mechanistic chain: the favorable hemodynamic environment induced by regular exercise is suggested to reinforce the vascular barrier and may contribute to the transition of atherosclerotic plaques toward a stable phenotype by targeting and remodeling the eGCX. This protective effect is hypothesized to involve three key mechanisms: first, the high-magnitude laminar shear stress induced by exercise promotes the synthesis and structural homeostasis of crucial glycocalyx components; second, functioning as the primary mechanosensory interface, the intact glycocalyx transmits beneficial shear signals to junctional complexes, stabilizing the cytoskeleton and intercellular junctions; third, the improved barrier function reduces endothelial permeability, thereby restricting the luminal influx of pathogenic lipoproteins and inflammatory mediators, which provides an essential microenvironmental foundation for fibrous cap repair and maintenance. This multi-level integrated mechanism driven by exercise shear stress has been systematically and visually summarized in Figure 1 of this review.

Although the aforementioned mechanistic framework is logically clear and possesses high biological plausibility, three critical evidence gaps remain in the current field: first, at the human pathophysiological level, causal evidence that exercise interventions alter local flow shear patterns and directly drive vulnerable plaque stabilization is still lacking; existing conclusions are mostly limited to extrapolations from animal models or other indirect evidence. Second, the quantitative criteria for the exercise-induced “shear dose” have not been clearly defined; the translational boundary between transient shear-induced glycocalyx shedding triggered by a single bout of strenuous exercise stress and structural reinforcement induced by long-term regular training remains unclear. Lastly, there is ongoing debate regarding the consistency between microvascular glycocalyx indices measured clinically (e.g., PBR) and the actual state of the glycocalyx at the sites of local vulnerable plaques in large arteries.

Directed towards deeper mechanistic exploration and clinical translation, future research should prioritize the following three optimized trajectories: first, regarding study design, computational fluid dynamics should be combined with high-resolution imaging to dynamically validate the effects of stage-specific shear stress responses on plaques; second, regarding endpoint evaluation, in addition to plaque burden, the synergistic remodeling of glycocalyx integrity, fibrous cap thickness, and the inflammation-protease network (MMP/TIMP) should be incorporated as core metrics; third, regarding population interventions, stratified studies targeting different disease states and individuals with distinct characteristics are needed to formulate personalized exercise prescriptions.

## Figures and Tables

**Table 1:** Approximate WSS ranges commonly referenced in atherosclerosis and flow-biology studies (values are context-dependent and vary by vascular bed, species, and measurement method).

Shear Stress Category	Typical Quantitative Range	Physiological/Experimental Context
Physiological/Atheroprotective WSS	>15 dyn/cm^2^ (Typically 15–70 dyn/cm^2^)	Characteristic of straight, unbranched segments of human large conduit arteries (e.g., normal aorta, carotid). Associated with quiescent, anti-inflammatory endothelial phenotypes.
Pathological Low WSS (In vivo)	<4 dyn/cm^2^ (often 0–4 dyn/cm^2^)	Found at arterial bifurcations, inner curves, and post-stenotic regions. Strongly correlates with plaque initiation and endothelial dysfunction.
“Low” WSS (In vitro models)	Highly variable (e.g., 2–12 dyn/cm^2^)	Experimentally defined relative to high-shear controls. Values like 12 dyn/cm^2^ may be “low” in specific flow chamber setups but remain within the normal physiological range for certain human vascular beds.
Pathologically Extreme High WSS	>70 dyn/cm^2^ (can exceed 100 dyn/cm^2^)	Localized at the throat or upstream shoulder of pre-existing severe stenotic plaques. Associated with fibrous cap thinning, high-risk remodeling, and plaque rupture.

(These ranges should not be interpreted as universal biological thresholds. 1 dyn/cm^2^ = 0.1 Pa).

**Table 2:** Exercise-related shear stimulus and endothelial adaptations: Representative evidence from human studies.

Study	Study Type/Subjects	Key Exposure or Intervention	Primary Endpoints	Core Conclusions
Tinken et al., 2010 [13]	Human training intervention study; healthy adults	Exercise training with manipulation of shear stress via localized cuffing	FMD, vascular remodeling	Endothelial adaptation induced by exercise was significantly attenuated when the shear stimulus was dampened, supporting that “shear stress is a key proximal stimulus for exercise-induced vascular adaptation.”
Birk et al., 2012 [32]	Human training study; observed upper limb conduit arteries following lower limb training	Shear stress associated with lower limb exercise training	Adaptive changes in the brachial artery	Supports that training-related hemodynamic stimuli can drive adaptive changes in conduit arteries.
Thijssen et al., 2009 [29]	Human experimental flow manipulation study	Enhanced retrograde flow/retrograde shear rate	Endothelial function	Retrograde shear acutely impairs endothelial function, suggesting that the “direction of shear” intrinsically possesses biological significance.
Simmons et al., 2011 [34]	Human acute exercise study	Retrograde shear changes during the onset and sustained phases of cycling	Dynamics of brachial artery retrograde shear rate	Retrograde shear during exercise is not constant; it declines with thermoregulatory vasodilation, demonstrating that the exercise shear waveform is dynamic.
Johnson et al., 2012 [36]	Human post-exercise flow manipulation study	Augmented oscillatory/retrograde shear post-exercise	FMD	If detrimental oscillatory/retrograde shear is superimposed after exercise, the benefits to endothelial function are attenuated.
Ghardashi Afousi et al., 2018 [37]	Human randomized controlled training study; patients with type 2 diabetes	12 weeks of low-volume high-intensity interval training (LV-HIIT) or continuous moderate-intensity training (CMIT), 3 times/week	Brachial artery FMD, artery diameter, antegrade/retrograde shear rate, oscillatory shear index (OSI), NOx	Compared with CMIT and controls, LV-HIIT more significantly improved FMD, increased antegrade shear, and decreased retrograde shear; both modalities reduced OSI, but NOx elevation was more pronounced in LV-HIIT, suggesting that LV-HIIT may more effectively optimize shear patterns and improve endothelial function.
Tanahashi et al., 2017 [38]	Human study (cross-sectional + intervention); middle-aged and older adults	12-week aerobic training (intervention portion)	Brachial artery antegrade/retrograde shear rate patterns, brachial artery intima-media thickness (IMT)	Aerobic training increased antegrade shear, decreased retrograde shear, and improved brachial IMT, suggesting that training-related shear pattern remodeling may participate in vascular wall adaptation.
Lyall et al., 2019 [39]	Human acute crossover study; healthy participants	Comparison of a single bout of continuous vs. interval exercise	In-exercise antegrade/retrograde shear rate, OSI, pre- and post-exercise FMD, circulating miR-21	Continuous and interval exercise generated distinct in-exercise shear patterns, but the magnitude of acute FMD improvement was similar.
Majerczak et al., 2017 [40]	Human pre-post controlled training study; healthy young men	20-week moderate-intensity endurance training	Serum glycocalyx damage markers (syndecan-1, heparan sulfate), oxidative stress indices, antioxidant defense-related markers	Long-term moderate-intensity endurance training reduced glycocalyx damage markers accompanied by attenuated oxidative stress, supporting its protective role in glycocalyx integrity.
Schmitz et al., 2019 [41]	Human pre-post controlled training study; healthy adults	4-week running-based HIIT	Sublingual microvascular glycocalyx barrier indices, including PBR, where higher PBR generally indicates reduced glycocalyx exclusion capacity, perfused vessel density, related miRNAs	HIIT was associated with improvements in microvascular glycocalyx-related indices, suggesting that short-term training can rapidly induce early vascular protective adaptations.
Fuchs et al., 2022 [42]	Human observational study; healthy adults	Single 10 km run	Sublingual microcirculation PBR, RBC filling percentage	No significant immediate changes in glycocalyx-related indices were observed after a single aerobic run, suggesting that acute effects may depend on the time window and measurement methods.

**Table 3:** Flow patterns, glycocalyx remodeling, endothelial transport, and protective transcriptional programs.

Study	Model/Subjects	Key Exposure or Condition	Primary Endpoints	Core Conclusions
Florian et al., 2003 [53]	Endothelial cells in vitro	Shear stimulus + heparan sulfate proteoglycan intervention	NO-related mechanical responses	Heparan sulfate proteoglycans are crucial mechanosensing elements on the endothelial surface, not merely passive overlays.
Mochizuki et al., 2003 [45]	Isolated canine femoral artery perfusion	Flow/shear stimulus + hyaluronidase pre-treatment	Shear-induced NO production	Hyaluronic acid-related glycosaminoglycans play a key role in detecting and amplifying flow shear to trigger endothelial NO release.
Tzima et al., 2005 [22]	Endothelial cells/animal mechanistic study	Fluid shear stimulus	Mechanosensory complex, downstream signaling	Identified the PECAM-1/VE-cadherin/VEGFR2 mechanosensory complex, establishing the “luminal force-to-junctional signaling” framework.
Zeng et al., 2013 [56]	Endothelial cells	Fluid shear stimulus	Clustering of glypican-1 and heparan sulfate	Shear can induce the rearrangement and clustering of key glycocalyx components within lipid rafts.
Zeng & Liu, 2016 [57]	Endothelial cells	Varied steady shear magnitudes	eNOS activation	Glypican-1 is involved in eNOS activation under different shear magnitudes, suggesting the glycocalyx also encodes “shear quality”.
Bai & Wang, 2014 [47]	Endothelial cells in vitro	Shear stimulus and post-removal recovery observation	Spatial distribution/rearrangement of glycocalyx	Shear stimulus can induce spatial redistribution of the glycocalyx, supporting that the glycocalyx is a dynamically remodeling interface rather than a static structure.
Wang et al., 2020 [21]	Endothelial cells + in vivo validation	Comparison of laminar vs. oscillatory flow	Glycocalyx structure, hyaluronic acid, HAS2 membrane localization, glucobiosynthesis	Laminar flow promotes surface hyaluronic acid maintenance and stabilizes glycocalyx structure via the KLF2–HAS2–glucobiosynthesis axis, whereas oscillatory flow impairs this process.
Yang et al., 2018 [51]	Endothelial cells	Low shear stress	Hyal2, LKB1/AMPK/NADPH oxidase, glycocalyx impairment	Low shear can promote glycocalyx damage via Hyal2-related pathways.
Kraehling et al., 2016 [73]	Endothelium/animal study	Mechanisms of LDL uptake and transendothelial transport	ALK1-mediated LDL uptake and transendothelial transport	Lipid entry into the intima does not necessitate “complete rupture” of the barrier; ALK1-mediated active LDL uptake and transport can occur preemptively.
Huang et al., 2019 [74]	Endothelium/mice/atherosclerosis study	SR-B1–DOCK4-mediated transendothelial LDL transport	LDL transport, atherosclerosis	The SR-B1–DOCK4 axis drives transendothelial LDL transport and promotes atherosclerosis.
Tamargo et al., 2024 [70]	Mice, HAECs, human arteries	Comparison of steady vs. disturbed flow	HEG1, KLF2/KLF4, permeability, adhesion	Steady flow maintains the protective endothelial phenotype and suppresses permeability/adhesion via the HEG1–KLF2/KLF4 axis.
Joshi et al., 2024 [71]	Mouse and human endothelial systems	Flow-sensitive transcriptional regulation	γ-protocadherins, KLF2/KLF4	γ-protocadherins can inhibit KLF2/KLF4 and promote atherosclerosis.

**Table 4:** Exercise training and downstream lesion/plaque phenotypes in atherosclerosis.

Study	Model/Subjects	Exercise Intervention	Primary Lesion/Plaque Endpoints	Core Conclusions	Level of Evidence
Kadoglou et al., 2011 [83]	ApoE-/- mice	Exercise training	Plaque area, collagen/elastin fibers, macrophages, MMP-9, TIMP-1	Exercise training attenuates lesion burden and improves plaque composition, associated with MMP inhibition, providing core direct evidence that “exercise improves plaque phenotypes.”	Animal model (in vivo)
Kadoglou et al., 2013 [81]	Diabetic atherosclerotic ApoE-/- mice	Exercise training	Plaque stabilization, inflammatory modulators, MMP-2/3, TIMP-2, fibrous cap phenotypes	Exercise training reduces and stabilizes atherosclerotic plaques in a diabetic context, suggesting its anti-inflammatory effects are closely linked to plaque stabilization.	Animal model (in vivo)
Cardinot et al., 2016 [88]	Atherosclerotic mouse models	Preventive or therapeutic moderate-intensity aerobic exercise	Plaque characteristics, collagen content, CD40–CD40L signaling	Moderate aerobic exercise converts plaques to a more stable phenotype, manifested by increased collagen, accompanied by decreased CD40–CD40L pathway activity in preventive protocols.	Animal model (in vivo)
Wu et al., 2019 [89]	ApoE-/- mice	Regular or occasional exercise (8 weeks)	Plaque burden, collagen, SMCs, lipids, macrophages, NPY/receptor expression	Exercise reduces plaque burden and enhances stability, shown by increased collagen/SMCs and decreased lipids/macrophages; this effect is at least partially related to the downregulation of NPY and its receptors.	Animal model (in vivo)
Stanton et al., 2022 [82]	Early and late-stage atherosclerotic ApoE-/- mice	10-week exercise intervention at different disease stages	Plaque stenosis degree, volume, composition, lipids, and matrix indices	Whether initiated at early or late stages, exercise mitigates lesion stenosis; earlier intervention yields more pronounced improvements in lipids and plaque composition.	Animal model (in vivo)
Xu et al., 2022 [90]	Western diet-induced ApoE-/- mice	12-week treadmill exercise	Aortic root/total aorta lesion area, lipid deposition, foam cells, BHB, cholesterol efflux proteins	Regular treadmill exercise reduces lipid deposition and foam cell formation, inhibiting atherosclerosis progression, associated with elevated serum β-hydroxybutyrate and enhanced cholesterol efflux.	Animal model (in vivo)
Huang et al., 2022 [91]	Western diet-induced ApoE-/- mice	Endurance exercise	Comprehensive atherosclerotic phenotypes, inflammation/chemokine signals, SCFAs	Endurance exercise mitigates Western diet-induced atherosclerosis, accompanied by improvements in obesity, inflammation, and chemokines; this effect is linked to gut microbiota and derived SCFAs.	Animal model (in vivo)
Wang et al., 2023 [92]	Obese WT and ApoE-/- mice	12-week swimming training; evaluation of exercise-derived skeletal muscle EVs	Atherosclerosis progression, PWV, metabolic phenotypes, EV-mediated effects	Exercise inhibits atherosclerosis progression in ApoE-/- mice; its protective effect at least partially relies on metabolic remodeling mediated by skeletal muscle-derived extracellular vesicles.	Animal model (in vivo)
Yang et al., 2023 [93]	Clinical cohorts and mouse models (including NEAT1-/-)	Exercise intervention	Atherosclerosis progression, endothelial pyroptosis, NEAT1/m6A/METTL14/KLF4-NLRP3 pathway	Exercise inhibits endothelial pyroptosis and delays atherosclerosis by downregulating NEAT1 and its m6A modification, providing evidence bridging “downstream lesion endpoints + endothelial mechanisms.”	Clinical cohorts and Animal model (in vivo)
Guo et al., 2025 [84]	ApoE-/- mice	10-week treadmill exercise	Lesion area, vascular inflammation, circulating exosomal let-7c-5p/TIMP-3/MMP-9 changes	Treadmill exercise mitigates atherosclerosis and vascular inflammation, offering a new translational mechanism via the downregulation of circulating exosomal let-7c-5p and upregulation of TIMP-3.	Animal model (in vivo)

## Data Availability

No new data were created or analyzed in this study. Data sharing is not applicable to this article.
